# Intrauterine programming of obesity and type 2 diabetes

**DOI:** 10.1007/s00125-019-4951-9

**Published:** 2019-08-27

**Authors:** Denise S. Fernandez-Twinn, Line Hjort, Boris Novakovic, Susan E. Ozanne, Richard Saffery

**Affiliations:** 1 Metabolic Research Laboratories and MRC Metabolic Diseases Unit, Wellcome Trust-MRC Institute of Metabolic Science, University of Cambridge, Addenbrooke’s Hospital, Level 4, Box 289, Addenbrooke’s Treatment Centre, Cambridge, CB2 0QQ UK; 2 Department of Endocrinology, the Diabetes and Bone-metabolic Research Unit, Rigshospitalet, Copenhagen, Denmark; 3 Department of Obstetrics, Center for Pregnant Women with Diabetes, Rigshospitalet, Copenhagen, Denmark; 4 Murdoch Children’s Research Institute, Royal Children’s Hospital, Flemington Road, Parkville, VIC 3052 Australia

**Keywords:** Developmental programming, Epigenetic variation, Intrauterine programming, Life course development, Maternal exposures, MicroRNAs, Obesity, Paternal exposures, Review, Type 2 diabetes

## Abstract

The type 2 diabetes epidemic and one of its predisposing factors, obesity, are major influences on global health and economic burden. It is accepted that genetics and the current environment contribute to this epidemic; however, in the last two decades, both human and animal studies have consolidated considerable evidence supporting the ‘developmental programming’ of these conditions, specifically by the intrauterine environment. Here, we review the various in utero exposures that are linked to offspring obesity and diabetes in later life, including epidemiological insights gained from natural historical events, such as the Dutch Hunger Winter, the Chinese famine and the more recent Quebec Ice Storm. We also describe the effects of gestational exposure to endocrine disruptors, maternal infection and smoking to the fetus in relation to metabolic programming. Causal evidence from animal studies, motivated by human observations, is also discussed, as well as some of the proposed underlying molecular mechanisms for developmental programming of obesity and type 2 diabetes, including epigenetics (e.g. DNA methylation and histone modifications) and microRNA interactions. Finally, we examine the effects of non-pharmacological interventions, such as improving maternal dietary habits and/or increasing physical activity, on the offspring epigenome and metabolic outcomes.

**Electronic supplementary material:**

The online version of this article (10.1007/s00125-019-4951-9) contains a slideset of the figures for download, which is available to authorised users.

## Intrauterine exposures and programming of type 2 diabetes and obesity

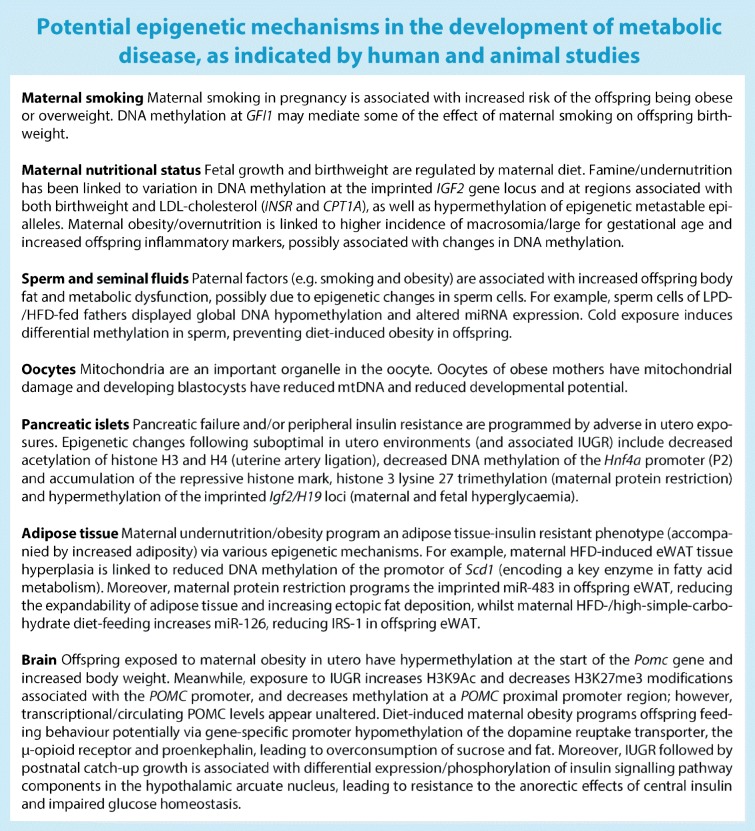



Aside from the direct influences of genetics and the environment on an individual’s propensity to develop obesity and type 2 diabetes, the last three decades have seen strong evidence to support the notion that many adult-onset diseases are linked to in utero exposures. Hales and Barker proposed the ‘thrifty phenotype hypothesis’ to explain how poor availability of nutrients or a poor diet in utero results in poor fetal growth and programs structural and metabolic responses in the developing fetus [1]. These responses would be advantageous if the nutritional environment is reflected postnatally, but potentially deleterious in an energy-rich postnatal setting. This hypothesis has evolved to encompass the periconceptional period through to infancy and describes how adverse exposures that occur at critical points of development may affect function and/or structure of an organ system into adulthood; it is now referred to as the Developmental Origins of Health and Disease (DOHaD) hypothesis.

### Fetal undernutrition and overnutrition

Fetal growth and birthweight are crude but commonly used measures of fetal wellbeing, shown to be regulated by maternal diet, lifestyle factors and the complex maternal–placental interplay [2]. Low birthweight is considered a marker for poor fetal nutritional status and has been associated with metabolic abnormalities, including type 2 diabetes and cardiovascular disease, in later life [3, 4]. Indeed, studies on prenatal famine during the Dutch Hunger Winter [5] showed that individuals exposed to famine while in utero exhibit decreased glucose tolerance some 50 years later compared with those born the year before the famine. Meanwhile, studies on adults born during the Chinese famine, between 1959 and 1961 [6], found that exposed individuals were more prone to be overweight and have type 2 diabetes, hyperglycaemia and the metabolic syndrome compared with those born after the famine. Additionally, interactions of the effects of the famine with an intergenerational risk of type 2 diabetes is cited as a major contributor to China’s current type 2 diabetes epidemic [7]. Twin studies support these findings: in monozygotic twin pairs discordant for type 2 diabetes, the twin with lower birthweight most often develops metabolic dysregulation [8]. Moreover, young adults with low birthweight display decreased muscle mass and height and increased fat mass compared with individuals of normal birthweight [4, 9]. Finally, more recent studies suggest that high birthweight is also associated with increased risk of obesity and type 2 diabetes [10]. This suggests that both fetal undernutrition and fetal overnutrition increase the risk of poor metabolic health later in life.

Fat and lean mass, both prenatally and in early postnatal life, also show relationships with in utero exposure, with a potential impact on future type 2 diabetes risk. For example, in a cohort of breastfeeding mother–infant dyads, in utero exposure to a higher maternal diet quality, based on the 2015 Healthy Eating Index (HEI-2015) [11], was inversely associated with infant body fat percentage [12]. Postnatally, breastfeeding or feeding a low-protein formula were associated with lower gain of fat mass (measured in children aged 5–8 years), whereas higher protein intake during the first 2 years postnatally resulted in higher BMI at 9 years of age and into adulthood [13].

### Infections and inflammation

Metabolic and immune pathways are extensively integrated in health and disease. Specific metabolites in the cholesterol and tricarboxylic acid (TCA) cycle have an effect on inflammation [14, 15], and, conversely, infectious diseases in pregnancy may contribute to developmental origins of metabolic conditions [16]. Viral infections in pregnancy, specifically by enteroviruses [17], have been associated with type 1 diabetes in the offspring, though the mechanisms are complex and evidence circumstantial [18, 19]. Two recent systematic reviews and meta-analyses identified a potentially causative link between maternal viral infections in pregnancy and type 1 diabetes in the offspring [20, 21]. Interestingly, monocytes from mothers with gestational diabetes show a proinflammatory profile [22], which can also be induced in fetal monocytes of mothers infected with hepatitis B virus [22]. Together, these studies highlight a close relationship between hyperglycaemia and inflammatory memory [23]. Infections in pregnancy, such as premature births with chorioamnionitis, have been associated with histone modification changes in cord-blood monocytes [24], and inflammation memory in vitro is epigenetically modulated [25] and reversible [26]. These findings indicate that infection in utero can alter epigenetic patterns in offspring cells, supporting a causal link between infection and offspring obesity, mediated by metabolic and epigenetic reprogramming.

### Environmental chemicals

Other prenatal exposures potentially linked to type 2 diabetes risk in later life include exposure to parental smoking [27, 28] and other environmental chemicals. For example, in utero exposure to dioxins, pesticides or bisphenol A in mice confers increased risk of developing type 2 diabetes [29]. In humans, exposure to organochlorines, as measured in second trimester maternal serum, was positively associated with BMI *z* scores and being overweight at 7 years of age [30]. Additionally, exposure to arsenic is linked to increased risk of gestational diabetes in the Maternal-Infant Research on Environmental Chemicals (MIREC) study [31] and in cohorts in France [32] and China [33], which poses an indirect threat to the affected offspring since gestational diabetes appears to be a programming factor for offspring metabolic dysfunction [34].

### Maternal stress

Effects of prenatal maternal stress have been studied in natural disaster cohorts, such as Project Ice Storm, which included individuals who were exposed to the Quebec Ice Storm [35]. In the children of mothers who experienced hardship and stress during the ice storm, the severity of stress predicted the levels of insulin [36] and C-peptide [37] secretion. Similarly, a Danish longitudinal study found that children who were prenatally exposed to bereavement were more likely to have a type 2 diabetes diagnosis later in life [38]. Prenatal stress has also been shown to increase rat offspring susceptibility to diet-induced obesity [39]. Maternal sleep fragmentation-induced stress in mice has also been shown to result in offspring metabolic disorders, including increased body weight, visceral fat mass and HOMA-IR [40]. It is likely that future studies will continue to identify additional early-life exposures that impact the risk of later-life obesity and type 2 diabetes.

### Gut microbiota

Disruption of the gut microbial community in newborns of obese mothers has also been shown to contribute to childhood inflammatory diseases, non-alcoholic fatty liver disease (NAFLD) and increased obesity risk [41]. This has been supported by studies showing that antibiotic use in the first year of life conferred an increased obesity risk [42], while synbiotics conferred protection against excessive fat accumulation under a high-fat diet (HFD) challenge [43]. In non-human primates, a maternal HFD was shown to reduce intestinal microbiota diversity in juvenile offspring at 1 year of age, even after switching to a healthy diet at the time of weaning [44].

### Paternal factors

Until recently, programming research has focused mainly on maternal exposures to programming. Although limited, there is evidence in humans to support programming of type 2 diabetes and obesity via paternal exposures; a paradigm coined the Paternal Origins of Health and Disease (POHaD) [45]. For example, paternal smoking has been associated with increased body fat in male offspring [46], while paternal obesity is associated with type 1 diabetes in offspring [47]. Evidence from animal studies is much stronger and will be discussed later in this review.

## Epigenetic mechanisms

Although the relationships between suboptimal in utero environments and increased risk of subsequent metabolic dysfunction are well established, underlying mechanisms have, until relatively recently, been poorly defined. In the last decade, numerous studies have implicated epigenetic mechanisms in the development of metabolic diseases through gene–environment interactions [48]. A range of exogenous exposures can influence epigenetic modifications, including the prenatal environment and adult lifestyle. Of particular note, compelling reproducible data have linked in utero exposure to smoking to defined changes in the offspring epigenome (see below).

Epigenetic mechanisms regulate gene activity in the absence of changes to the underlying DNA sequence, hence the name: ‘epi’, meaning ‘above’ in Greek, and ‘genetics’ [49]. Epigenetic mechanisms include DNA methylation, histone variants/modifications, chromatin-modifying proteins and non-coding RNAs. These processes regulate how densely specific regions of DNA are compacted, thus either inhibiting or enabling access of proteins, such as transcription factors, to DNA [50].

### DNA methylation/demethylation

DNA methylation is the most studied epigenetic feature, primarily because its covalent chemical structure makes it highly stable and, therefore, quantifiable in a range of archived tissue and cells. DNA methylation is dispersed at varying densities across the genome, with specific variations of the methylation pattern being linked to cell identity and function [51]. In higher animal species, including humans, the main target is cytosines in CG dinucleotides, also referred to as CpG sites [52]. One feature of the vertebrate DNA methylation profile is the presence of CpG islands, regions of high-density CpG sites, located near or in gene-promoter regions. Around 29,000 CpG islands have been identified in the human genome [53]. DNA methylation in promoter regions may induce transcriptional inhibition or repression by affecting transcription-factor binding or recruiting proteins that specifically bind to methylated CpG sites [54].

DNA methyltransferases (DNMTs) transfer a methyl group to the 5′ position of cytosine. DNMT1, the maintenance methyltransferase, copies methylation status of hemimethylated sites after cell division [51]. In contrast DNMT3A and DNMT3B carry out de novo DNA methylation of unmethylated DNA, particularly in early embryonic development [55].

**Fig. 1:**
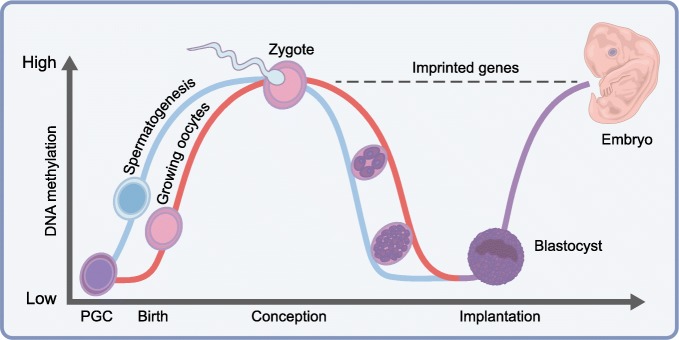
DNA methylation dynamics during human development. Male (blue line) and female (red line) embryos follow different DNA methylation patterns, from the birth of the parent through to zygote production (conception) and blastocyst implantation. Imprinted genes (dashed black line) do not undergo demethylation post-fertilisation and, hence, reflect parental-allele-specific methylation. PGC, primordial germ cells. Adapted from [125], with permission from Elsevier. This figure is available as part of a downloadable slideset

Demethylation can be a passive process, such as cell division without maintenance by DNMT1, or actively carried out by several enzymes, including the methylcytosine dioxygenases (ten-eleven translocation [TET]) enzymes that oxidise 5-methylcytosine (5meC) to 5-hydroxy methylcytosine (5hyroxy-meC) and other derivatives [56]. Each of these demethylation processes are important for appropriate gene expression and cell specification, particularly during early preimplantation development, as shown in Fig. 1 [57].

Although less dynamic, methylation also changes throughout postnatal life and adulthood. It is estimated that methylation of approximately 30% of all methylated sites in leucocytes or whole blood changes in an age-dependent manner [58, 59]. The methylation status of blood cells has also recently been shown to mirror age-related epigenetic signatures in adipose tissue [59]*.* Therefore, diet and other environmental factors throughout childhood and adulthood should also be considered when investigating epigenetic mechanisms in birth cohort studies of long-term health, since some epigenetic markers at specific loci appear to be much more flexible compared with those reported as stable markers over the long term [3].

The number of ‘epigenetic epidemiology’ papers and ‘epigenome-wide association studies’ (EWAS) published has increased sharply in the last 6 years and, coupled with locus- (gene-)specific epigenetic–environment studies, a plethora of data has emerged [60]. Despite good-practice approaches, such as publishing primer sequences and correctly referencing genome builds and CpG site locations, comparisons between datasets is not always straightforward, especially regarding the interpretation of what the ‘functional’ consequence of a change in DNA methylation means. For example, different quantification techniques have vastly different sensitivities and, therefore, some can detect small changes in DNA methylation (e.g. Infinium HumanMethylation arrays or targeted bisulphite sequencing), while others cannot (e.g. whole-genome bisulphite sequencing or bisulphite cloning and sequencing) [61]. Second, while genome-wide association studies (GWAS) studies can be carried out on any available cell type, DNA methylation varies between cell types and studies that use whole tissues or whole blood need to use algorithms to account for different cell types [62].

### Histone modifications

Histone modifications occur in the N-terminal tail domains, in the core histone domains and in newly synthesised histones. Histone tails contain numerous sites that are amenable to acetylation and phosphorylation, which can alter the charge of the tails, thus affecting chromatin architecture through electrostatic mechanisms. These modifications act as ‘docking’ sites for chromatin ‘readers’ that recognise these modifications and recruit additional chromatin modifiers and remodelling enzymes [63]. It is now widely accepted that acetylation of histones inhibits the secondary and tertiary nucleosome structure, resulting in chromatin decondensation and increasing access to transcription factors and co-activators of transcription. In contrast, histone methylation has opposing effects, causing nucleosomal arrays to fold and condense, thus allowing active transcription [64].

### microRNAs and long non-coding RNAs

Yet another regulatory mechanism contributing to phenotypic variation can occur at the post-transcriptional and transcriptional level; the emerging components of this type of regulation are microRNAs (miRNAs), which are small (21–24 nucleotide long) molecules that bind specifically to the 3′ untranslated regions of mRNA and interact with the Dicer complex. This binding sequesters the mRNA for degradation or prevents its translation by interfering with translation machinery. Additionally, long non-coding RNAs **(**lncRNAs) can bind mRNAs and act as molecular ‘sponges’ with opposing roles in transcript stabilisation/destabilisation. The roles of these two regulatory systems in type 2 diabetes pathogenesis has recently been reviewed by Saeedi et al [65].

## Epigenetic variation in utero and metabolic programming

### Maternal exposures

Several EWAS studies have found an association between maternal smoking and altered DNA methylation in cord blood [66], an effect that can persist postnatally [67] and into adulthood [68]. Maternal smoking has previously been linked to offspring obesity, with a linear dose-dependent effect, plateauing at 15 cigarettes or more per day [69]. Data from multiple studies and meta-analyses suggest a causative link between maternal smoking and increased risk of obese or overweight offspring [70]. Importantly DNA methylation at a specific gene, *GFI1*, was shown to mediate the effect of maternal smoking on offspring birthweight, explaining up to 19% of the difference in birthweight between offspring from mothers who smoked or did not smoke (control group) during pregnancy [71].

### Maternal nutritional status and epigenetics

In the first study examining the effect of the Dutch Hunger Winter on epigenetic marks (i.e. chemical modifications on the DNA sequence), individuals who were 60 years old and prenatally exposed to this famine were found to have less DNA methylation at the imprinted *IGF2* gene locus compared with their unexposed same-sex siblings [3]. More recently, genome-scale analysis in whole blood from this cohort identified that prenatal malnutrition-associated differentially methylated regions (P-DMRs) preferentially occurred at regulatory regions and were characterised by differential DNA methylation at regions associated with birthweight and serum LDL-cholesterol, i.e. *INSR* and *CPT1A* [72]. Hence, differential methylation of the P-DMRs extends along pathways related to growth and metabolism. Further exploratory analysis of six P-DMRs showed that they do not overlap with previously published adult tissue-specific differentially methylated regions (DMRs), highlighting that their establishment is dependent on specific exposure to famine during gestation.

Further evidence for a role of maternal nutrition in regulating the offspring epigenome comes from the Kiang West Longitudinal Population Study (KWLPS) [73], which included a cohort of 14,000 individuals from The Gambia that were subject to two distinct seasons, a hot dry ‘harvest’ season associated with high food abundance and a wet ‘hungry’ season associated with low energy intake [74]. Residents born in the hungry season were more likely to die prematurely (before the age of 25 years) [75] and to be small for gestational age [76]. Targeted epigenetic metastable epialleles, which are genomic regions that show significant inter-individual variation in DNA methylation in the absence of a genetic difference [77], were generally hypermethylated in individuals conceived during the hungry season, possibly as a result of increased one-carbon donor concentrations in the mother during this period [78]. Subsequent studies showed that multiple one-carbon donors, folate, riboflavin, betaine and choline all showed season-specific variation and their plasma concentrations predicted DNA methylation levels at metastable epialleles [79, 80]. The KWLPS cohort was used in conjunction with other datasets to identify a novel obesity-related (-predictive) metastable epiallele at the gene encoding pro-opiomelanocortin (POMC), which is similarly affected by maternal one-carbon donor concentration at conception [81].

### Maternal overnutrition/obesity

The incidence of maternal obesity at conception and in pregnancy is increasing [82] and there is evidence that it contributes to increased infant birthweight (macrosomia and large for gestational age) and higher BMI in adolescent offspring [83, 84]. Excessive gestational weight gain (GWG) during pregnancy is also associated with increased offspring BMI and inflammatory markers (IL-6 and C-reactive protein), with early-gestation GWG having a stronger effect on offspring BMI at age 5 years and adiposity at age 9 years than mid–late-gestation GWG [85, 86]. Interestingly, while GWG in all trimesters affects birthweight, only first-trimester GWG affects child weight gain, suggesting that moderation during the first trimester may have the biggest impact on childhood weight [86].

Epigenetics are thought to mediate these effects, prompting several studies into the DNA methylation changes associated with maternal obesity [87–89]. Maternal diabetes correlates with obesity; in these studies, it was either removed as a covariate [88], was considered indistinguishable from obesity [87] or the cohort was structured to only contain pregnant women with obesity but not diabetes [88]. Two epigenome-wide studies analysed blood from the umbilical cord of offspring, and from 4–5-year-olds and 9–16-year-olds [87], who were exposed to maternal obesity (with or without gestational diabetes) and identified many differentially methylated sites in exposed offspring. Despite the relatively modest effect (generally <5% change), such data suggest that maternal obesity can lead to DNA methylation changes that are present at birth and remain postnatally.

## Animal studies

Much of our understanding of programmed metabolic disease comes from animal models of under- and overnutrition. Studies in models of both ends of the nutritional spectrum have sought to understand potential programming mechanisms of type 2 diabetes and obesity risk by exploring epigenetic changes throughout the life course of exposed offspring. Importantly, unlike human studies, animal models allow the direct assessment of molecular and cellular defects.

### Sperm and seminal fluid

Paternal low-protein diet (LPD) has been shown to enhance offspring fetal growth and predisposition to increased adiposity, glucose intolerance and cardiovascular dysfunction in the adult [90, 91], with both sperm and seminal fluid of LPD-fed fathers exerting programming effects. Similarly, diet-induced obesity in the father programs an impaired metabolic profile in his offspring, [92, 93]. In both fathers who were LPD and HFD fed [94], sperm cells displayed global DNA hypomethylation and altered miRNA expression. Aside from diet-induced programming, cold exposure has also been shown to induce differential methylation in sperm, which conferred enhanced brown adipose tissue (BAT) activity and protection from diet-induced obesity in male offspring [95]. In parallel, it was observed in humans that the presence of BAT and the season of conception were linked to offspring BMI.

### Oocytes

Mitochondria are the most important organelle in the oocyte. While somatic cells maintain a healthy population of mitochondria by mitophagy, this pathway may not be active in oocytes. Thus, mitochondria damage in these germ cells may be transmitted to the developing blastocyst [96]. Such mitochondrial damage has been shown to occur in oocytes of obese dams, which have reduced mitochondrial DNA (mtDNA), accumulate the mitophagy marker phosphatase and tensin homologue-induced kinase 1 (PINK1) and demonstrate reduced developmental potential. The developing blastocysts show reduced levels of mtDNA and parallel mitochondrial loss in offspring that is caused by endoplasmic reticulum (ER) stress and which is reversible by ER stress inhibitors [97].

### Pancreatic islets

Pancreatic failure and/or peripheral tissue insulin resistance are both programmed by adverse in utero exposures. Islet transcription factors are vulnerable to epigenetic changes as a response to suboptimal in utero environments leading to intrauterine growth restriction (IUGR). Uterine artery ligation in rats led to decreased histone H3 and H4 acetylation and loss-of-binding of the upstream stimulatory factor 1 (USF-1) transcription factor to the proximal promoter of *Pdx1* in pancreatic islets, resulting in its markedly reduced transcription [98]. Maternal protein restriction in rats also led to reduced expression of *Hnf4a* in pancreatic islets of young male offspring in adulthood, which was associated with increased DNA methylation at the active *Hnf4a* promoter (P2) and increased repression through histone methylation at the enhancer region of this gene [99]. Consistently, the P2–enhancer interaction in islets of affected male offspring was significantly reduced, providing a mechanistic basis for reduced *Hnf4a* expression. Furthermore, the repressive histone mark, histone 3 lysine 27 trimethylation (H3K27me3), was found to accumulate with age in programmed offspring islets [99]. While insulin resistance was also observed in the female offspring in this model of IUGR, this was only evident in older mice [100]. Changes in DNA methylation have also been observed in pancreatic islets from a mouse model of maternal and fetal hyperglycaemia. Hypermethylation of the imprinted *Igf2/H19* loci in pancreatic islets was observed and proposed to drive impaired islet structure and function [101] and, at the age studied, impaired glucose tolerance was more evident in male offspring than in females and accompanied by male-specific transmission to the next generation.

### Adipose tissue

Adipose tissue has been shown to be an important target of developmental programming in animal models of both maternal undernutrition and overnutrition. In studies carried out only in male offspring and, specifically, in the epididymal white adipose tissue (eWAT), both maternal undernutrition [102] and maternal obesity [103] program an adipose tissue-insulin resistant phenotype accompanied by increased adiposity [102, 104, 105]. These programmed changes have both been attributed to epigenetic changes in adipose tissue. In addition, eWAT tissue hyperplasia due to maternal high-fat feeding during lactation was associated with increased expression and activity of stearoyl-CoA desaturase-1 (SCD1), a key enzyme in fatty acid metabolism. Changes in the expression of this enzyme were related to reduced DNA methylation of the *Scd1* promoter [106].

Programmed changes in miRNAs have also been implicated in the programming of both adipose tissue expandability and insulin resistance. For example, using a rat model of maternal protein restriction, it has been shown that the imprinted miR-483 is programmed in eWAT of male offspring [107]. This was accompanied by a reduction in the expression of its direct target, *Gdf3*, and a reduction in the expandability of adipose tissue and, therefore, increased ectopic fat deposition, which is a major contributor to the development of insulin resistance. Importantly, an increase in adipose tissue miR-483 and parallel reduction in growth differentiation factor 3 (GDF-3) was also observed in adipose tissue from humans with low birthweight, showing conservation of this programmed mechanism. Programmed changes in miRNAs were also observed in a mouse model of maternal diet-induced obesity [103, 105]. Maternal feeding of a high-fat and high-simple-carbohydrate diet led to a programmed increase in miR-126, which led to a reduction in its direct target, insulin receptor substrate-1 (IRS-1), in eWAT of male offspring [103]. This programming effect was cell autonomous and was maintained in cultured pre-adipocytes differentiated in vitro*,* demonstrating that it was related to the programming of the adipocyte precursor stem cell pool.

### Brain

The intrauterine environment also imposes important programming effects on the developing brain. Hypermethylation within a 500 bp region of the translation initiation start of the *Pomc* gene was observed in female offspring (Wistar outbred rats) exposed to maternal obesity in utero, corresponding with decreased *Pomc* transcription and increased body weight [108]. Diet-induced maternal obesity has also been shown to program feeding behaviour in the offspring by altering dopamine and opioid-related gene expression within the mesocorticolimbic reward pathways and hypothalamus [109]. This was linked to gene-specific promoter hypomethylation of the dopamine reuptake transporter, the μ-opioid receptor and proenkephalin, leading to an increased preference for sucrose and fat. The effects of overconsumption of these highly palatable and energy-dense foods are associated with obesity.

Conversely, in sheep, exposure to IUGR resulted in increased H3 lysine 9 acetylation (H3K9Ac) and decreased H3K27me3 modifications associated with the *POMC* promoter, and decreased methylation at a *POMC* proximal promoter region. However, these were not associated with either transcriptional or circulating POMC levels [110, 111]. In male C57BL/6J mice with IUGR followed by postnatal catch-up growth, differential expression and phosphorylation of components of the insulin signalling pathway in the arcuate nucleus of the hypothalamus effectively contributed to resistance to the anorectic effects of central insulin and impaired glucose homeostasis [112].

## The importance of intervention studies

### Lifestyle: diet and physical activity

The influence of dietary factors on both epigenetic patterns and phenotype provides a possible link between epigenetic marks and human metabolism.

Certain nutrients function as substrates for epigenetic modifications or co-factors for epigenetic enzymes and, therefore, diet can influence epigenetic patterns by varying epigenetic substrate availability or by altering the activity of enzymes that are involved in the addition or removal of epigenetic marks. A well-studied example is S-adenosyl methionine (SAM), a methyl donor substrate that provides methyl groups to both DNA and histone methyltransferases [113].

One of the strongest examples of an epigenetic alteration in adulthood, which is caused by environmental exposures during prenatal, childhood or adult life, is promoter DNA methylation of the key metabolic regulator, peroxisome proliferator-activated receptor, gamma, coactivator 1, alpha (PGC-1α) (encoded by *PPARGC1A*). PGC-1α plays a role in the regulation of genes required for energy metabolism, mitochondrial biogenesis and adaptive thermogenesis [114]. *PPARGC1A* expression is down-regulated in skeletal muscle from individuals that have impaired glucose tolerance or diabetes [115], while healthy men exposed to a high-fat overfeeding (HFO) diet for 5 days show increased DNA methylation at the *PPARGC1A* promoter in both adipose tissue and skeletal muscle [116, 117]. Feeding status has also been shown to affect methylation state; for example, 36 h of fasting affected DNA methylation of genes encoding leptin (*LEP*) and adiponectin (*ADIPOQ*) in adipose tissue [118].

Regular exercise has also been associated with wide-spread DNA methylation changes in a variety of tissues [119]. On the other hand, sedentary behaviour (9 days of bed rest) in healthy young men resulted in increased *PPARGC1A* DNA methylation and decreased *PPARGC1A* gene expression in skeletal muscle [120]. Recent data also shows that exercise may regulate histone deacetylases (HDAC) that further induce the expression of genes that play a role in metabolic pathways [121]. All in all, evidence suggests that a sedentary lifestyle can lead to genome-wide epigenetic changes and that physical exercise could be a possible mechanism to reverse these changes.

The benefits of increased physical activity have been interrogated in animal obesity or HFD-feeding models of programmed disease. In murine models of maternal obesity, daily treadmill running for 1 week prior to and throughout gestation led to improved insulin sensitivity in young adult offspring, which was associated with prevention of the programmed reduction in adipose tissue IRS-1 [122]. These exercise-driven improvements were analogous to those observed in 1-year-old offspring of mothers fed a HFD and housed with running wheels [123, 124]. The same research group showed similar benefits to 1-year-old offspring of HFD-fed fathers that had been exposed to voluntary exercise [92].

## Conclusions and future perspectives

**Fig. 2:**
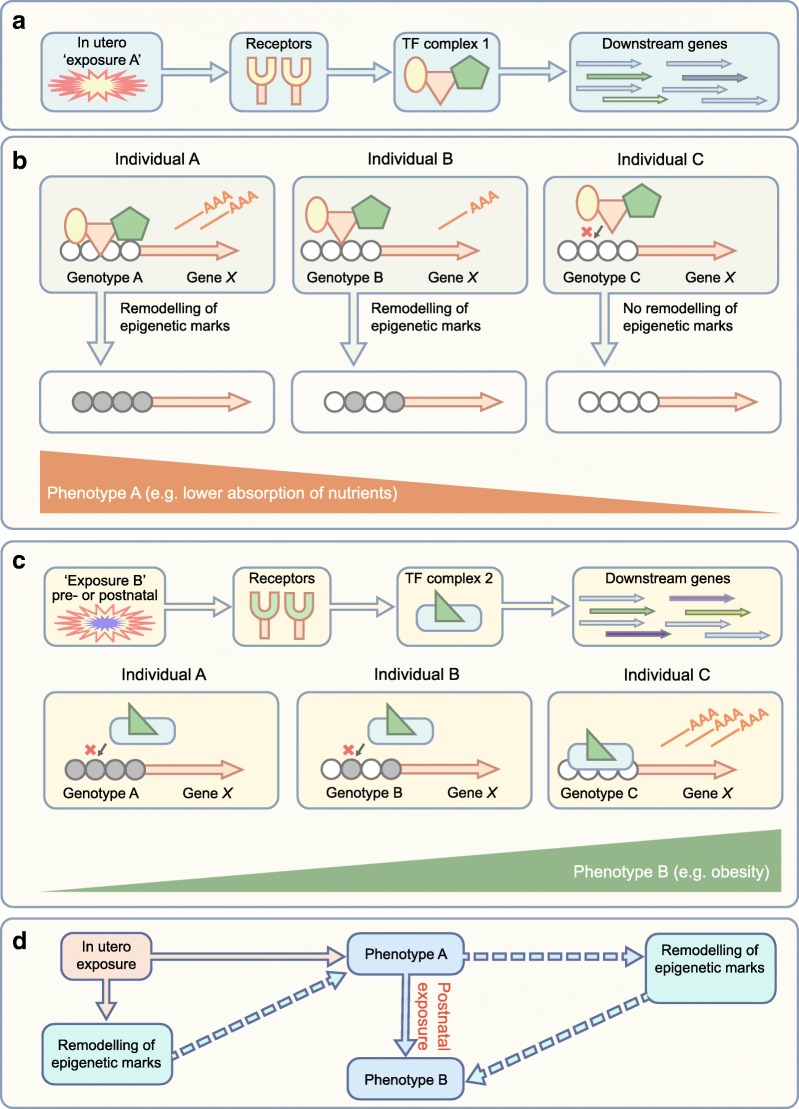
A complex interplay between in utero environment, genetics, epigenetic marks and phenotype. (**a**) The fetus is exposed to various maternal exposures in utero, some of which are potentially detrimental, leading to activation of transcriptional regulators (via receptors) and of downstream genes. (**b**) Genetic differences also influence transcription factor binding and regulation of downstream gene expression (mRNA shown as ‘——AAA’). Transcription factor binding can lead to recruitment of epigenetic modifiers, reprogramming epigenetic marks at gene-regulatory elements. Variation in these epigenetic marks (e.g. DNA methylation, shown as grey shading of circles) often correlate with specific phenotypes at birth. (**c**) During postnatal life, individuals are continually exposed to environmental exposures that further modulate gene expression and phenotype. Differential epigenetic reprogramming in utero can affect the transcriptional response of cells to these new exposures, leading to different adult phenotypes. In the illustrated example, epigenetic remodelling of metabolism/nutrition genes during in utero development can lead to adult obesity. (**d**) A model showing how an initial exposure can alter the phenotype, leading to susceptibility to disease in response to a second environmental exposure. Solid lines indicate the order of events leading to phenotype B, while the dashed lines indicate the potential, but unproven, role for epigenetic variation in contributing to the phenotypes. The illustrations in (**a**–**c**) are based on decades of research showing that epigenetic marks can mediate the effect of exogenous signals on gene expression and phenotype. Nevertheless, showing causality is often difficult in humans and, in some cases, epigenetic differences may only correlate with exposure and outcome, but not necessarily contribute to phenotype. Longitudinal studies that make use of samples collected before disease onset are essential to tease out these causal/passenger questions. TF, transcription factor. This figure is available as part of a downloadable slideset

There is now compelling evidence for the transmission of poor metabolic health across generations. Mounting evidence shows that specific in utero environments (exposures) can have an impact on offspring epigenetic profile in a manner that is stable postnatally, into adulthood, in association with changed phenotype (Fig. 2). Despite these compelling data, only limited evidence exists for a causal role for epigenetic variation in mediating the effects of adverse in utero environment(s) on poor offspring metabolic health. Further additional longitudinal human studies are urgently needed to build this evidence base, supplemented with ongoing animal model studies that allow direct assessments of target tissues of relevance. Such a complementary approach should reveal the extent to which variation in epigenetic profile might act as a predictive early-life biomarker of increased metabolic risk, enabling targeting of novel interventions to those most likely to benefit. Further, the considerable interest in developing therapeutic epigenetic-modifying drugs and the increasing knowledge about the epigenetic-modifying properties of many dietary factors represent likely future approaches for modifying and reversing adverse metabolic health trajectories by (nutri) pharmacogenomic approaches.

## Electronic supplementary material

Slideset of figures (PPTX 343 kb)

## Abbreviations

BAT: Brown adipose tissue
DNMT: DNA methyltransferase
ER: Endoplasmic reticulum
EWAS: Epigenome-wide association studies
eWAT: Epididymal white adipose tissue
GWG: Gestational weight gain
HFD: High-fat diet
H3K27me3: Histone 3 lysine 27 trimethylation
IUGR: Intrauterine growth restriction
KWLPS: Kiang West Longitudinal Population Study
LPD: Low-protein diet
miRNA: MicroRNAs
mtDNA: Mitochondrial DNA
P-DMR: Prenatal malnutrition-associated differentially methylated region
PGC-1α: Peroxisome proliferator-activated receptor, gamma, coactivator 1, alpha
POMC: Pro-opiomelanocortin
