# Supplementary material for: Intrauterine programming of obesity and type 2 diabetes
Source: Diabetologia. 2019 Aug 27;62(10):1789–801. doi: 10.1007/s00125-019-4951-9 (PMC6731191; doi:10.1007/s00125-019-4951-9)
Supplement: Supplementary file 1 — (PPTX 343 kb) [file 125_2019_4951_MOESM1_ESM.pptx]

## Slide 1
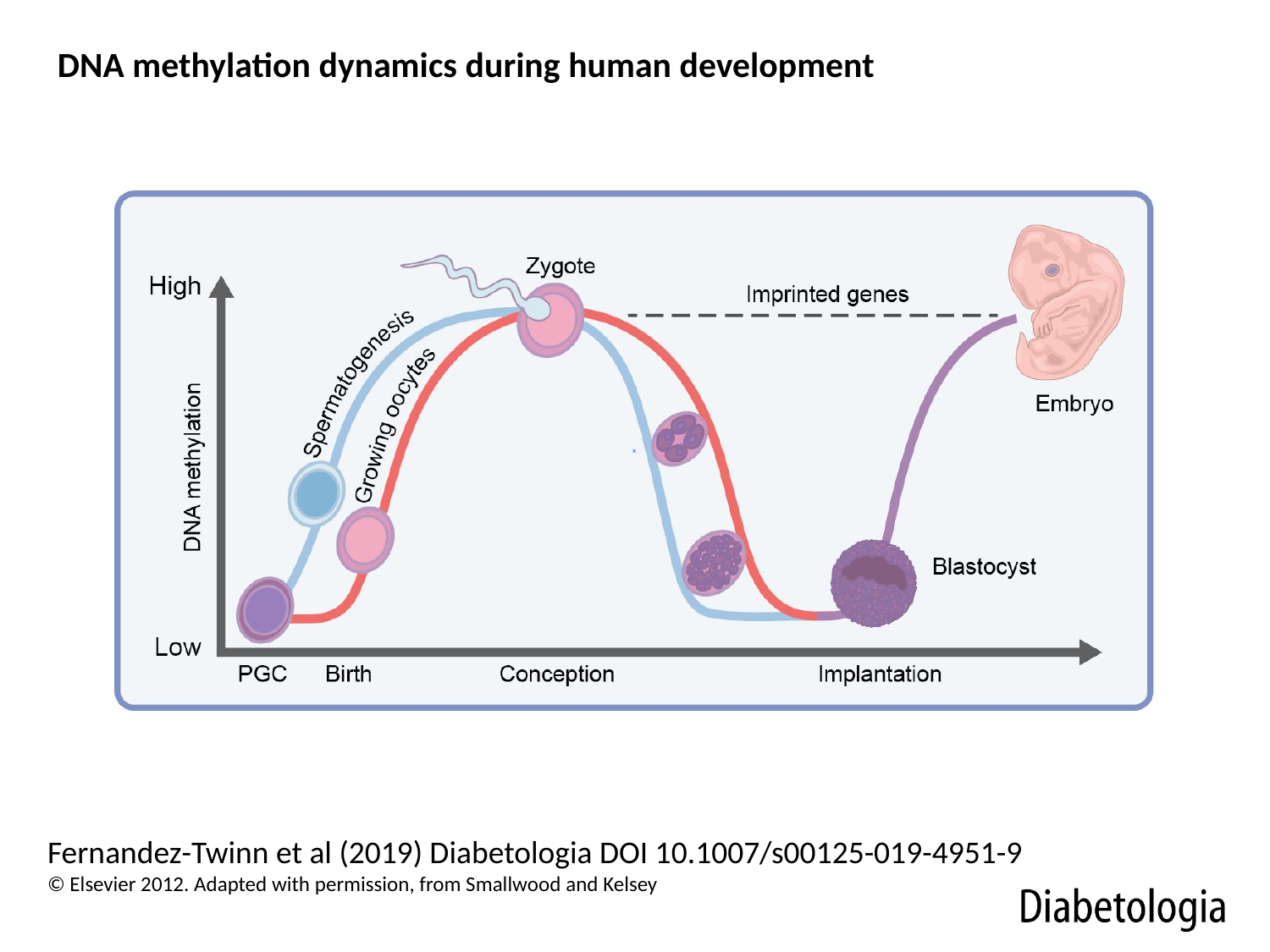

DNA methylation dynamics during human development
Fernandez-Twinn et al (2019) Diabetologia DOI 10.1007/s00125-019-4951-9
© Elsevier 2012. Adapted with permission, from Smallwood and Kelsey

## Slide 2
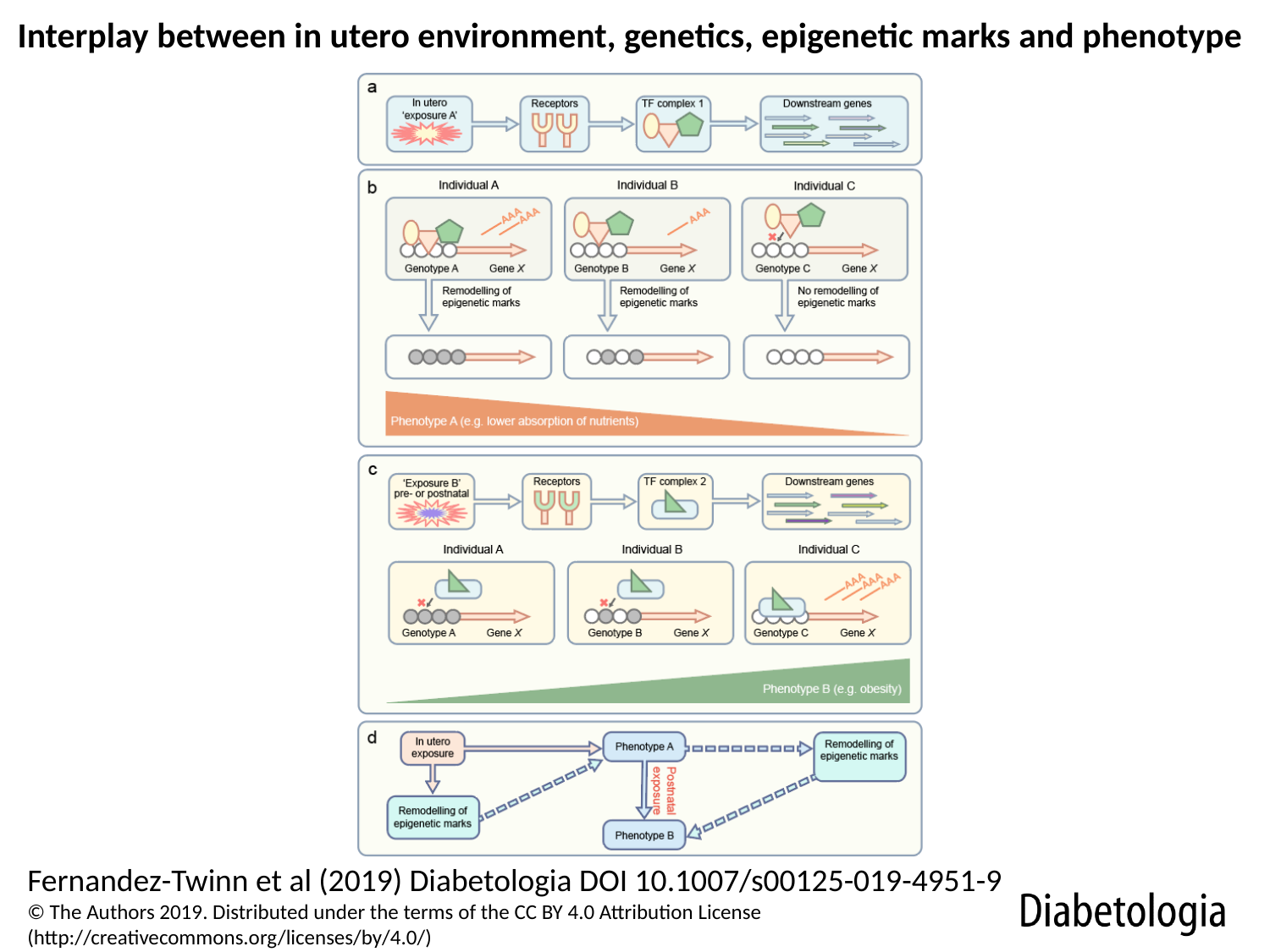

Interplay between in utero environment, genetics, epigenetic marks and phenotype
Fernandez-Twinn et al (2019) Diabetologia DOI 10.1007/s00125-019-4951-9
© The Authors 2019. Distributed under the terms of the CC BY 4.0 Attribution License
(http://creativecommons.org/licenses/by/4.0/)
